# Differences in the Bodyweight, Hydration Levels, Lean Mass, and Fat Mass in Spanish Junior Elite Judokas

**DOI:** 10.3390/ijerph17082853

**Published:** 2020-04-21

**Authors:** David Gamero-delCastillo, Jorge Lorenzo Calvo, Archit Navandar, Alfonso López Díaz de Durana

**Affiliations:** 1 Sports Department, Faculty of Physical Activity and Sports Science—INEF, Universidad Politécnica de Madrid, 28040 Madrid, Spain; Archit.navandar@upm.es (A.N.); alfonsolopezdiazdedurana@gmail.com (A.L.D.d.D.); 2 AFIPE Research Group—Sport Physical Activity in Specific Populations, Faculty of Physical Activity and Sports Science—INEF, Universidad Politécnica de Madrid, 28040 Madrid, Spain

**Keywords:** combat sport, judo, body composition, dehydration, bioimpedance, weight loss

## Abstract

Combat sports have a great interest in society and among professional sports. They are an important group of sports in the Olympic Games, but the strategies carried out by athletes to reduce body weight for weighing day, is famously known, suffering the adverse physical and psychological effects of rapid weight loss. This could compromise not only the performance, but the health and development of young athletes. A total of 22 elite male judokas (18.05 ± 1.05 years old) were evaluated during four different competitions in one season; the variables of body weight, water levels, and lean and fat mass were measured by bioimpedance (BIA), (Tanita BC545N) during one season. Using the linear mixed model test, we found significant differences in bodyweight variable during the competitions 3–4. The water level variable showed significant differences in all competitions, except for 2–4. Body mass index was significantly different in all competitions, being higher in the later competitions, except between competitions 1–2 and 2–3. Judokas participate in weight loss methods for their weigh-in days. Furthermore, the age at which the athletes reduced their water levels are worrisome. These results could be used to create healthy programs, especially in elite judokas, in order to carry out strategies before, during, and after competitions with weight loss and controlled water levels increasing future performance and health.

## 1. Introduction

Combat sports represent 25% of Olympic medals, athletes are classified according to their weight in these sports. Many of them reduce their body mass drastically to gain advantage and compete against lighter and smaller opponents [1], because there is a belief that lighter athletes have a higher strength–body weight ratio [2]. It is difficult to discourage athletes from these practices, but they can be made aware, if there is evidence that such practices could affect their muscular functioning [3]. To explain this muscular damage, studies have been carried out on elite fighters and in healthy elite and amateur athletes who were evaluated after intensive exercise until muscular exhaustion, causing an elevation of cardiac biomarkers in 62% of the subjects like troponin, a possible indicator of heart damage, acute coronary syndrome, or even infarction [4,5,6,7,8,9,10,11]. Wrestlers were divided into groups, depending on their hydration levels, and it was concluded that the levels of inflammation and markers of muscle damage (creatine phosphokinase, CPK, lactate dehydrogenase, LDH, and aspartate transaminase, AST) obtained in their post-competition blood samples were different between both groups and higher in the forearm muscles of the dehydrated group. In addition, there is greater evidence of the adverse effects of rapid weight loss on health status [5]. The negative effects of fast weight loss on the physical and psychological health of athletes have been studied by several authors. This behavior significantly reduces lean mass [12,13,14], reserves of liquids and carbohydrates [15], impairs immune function [16], decreases vigor, and increases fatigue, anger, anxiety, tension, and confusion [17]. Other studies [18] have indicated that mild dehydration (1% Bw (bodyweight)-induced by exercise) affects eye care and response and increases fatigue, temperature, anxiety, and stress. In judo, the percentage of athletes who reduce their weight was 62.8%, rapid weight loss was practiced by 80% of younger judo athletes before competitions, and beginning to practice RWL at 12.5 ± 2.2 years, with the highest prevalence (~94%) in the group of judokas who were 16–17.9 years old, according to [5,6,7]. These values are like the percentages for Jiu Jitsu (56.8%), Karate (70.8%), and Taekwondo (63.3%).

Body mass loss requires weeks of diet, and the loss of carbohydrates stored in the body should be only −0.5 kg. Therefore, most of the weight loss achieved by combat sports athletes is due to body water, with extreme reductions in the intake of fluids [3]. This reveals the importance of understanding the role of body fat percentage in energy management for training and competitions. In judoka of a high level, previous studies have already determined the mean and standard deviation of body fat (Table 1).

In relation to hydration, a difference must be established between hyperhydration and hypohydration. The term hypohydration refers to a state of suboptimal hydration, and dehydration describes the transition from a well hydrated a hypo hydrated state [25].

In addition, techniques used for the autoinduction of dehydration include exposure to a sauna and endurance exercises, or disorders associated with corporal weight. Other more aggressive techniques include vomiting, diuretics, laxatives, or anorexic medication [26], which are the preliminary steps in developing ED (eating disorders), such as anorexia and nervous-type bulimia.

ED are serious alterations, characterized by a disproportionate and abnormal consumption of foods and nutrients, in order to modify body weight. Consequently, ED have a negative impact on physical and psychosocial health [26,27,28].

These ED can also affect athletes at the psychobiological level, with abnormal behaviors due to an equivocal perception of size and body image, which is distorted [29,30]. The prevalence of ED among Judoka is 35.7% for both sexes. Anorexia nervosa has a prevalence of 2.4% in the samples studied and is more common among judoka of the female sex than among male judoka (54.8%–45.2%) [28].

The use of the mentioned techniques over the days before the weigh-in could lead to progressive dehydration because many athletes are not properly rehydrated. After 2–3 days of dehydration, intracellular fluids need 48 h to be replaced and available. A state of dehydration leads to a decrease in strength, power, and endurance in high intensity exercises, thus negatively affecting thermoregulation and increasing body temperature and susceptibility to excess heat diseases [8]. Dehydrated athletes often experience cognitive changes and altered mental states [9,25]. A water loss of 2% body weight can compromise cardiorespiratory resistance and muscular function, thus affecting performance [8].

Although most studies of the effects of dehydration on performance have included only adult participants, dehydration is also detrimental to aerobic performance and skill-based activities among children athletes [8,25]. The most tragic consequence of dehydration was the death of three wrestlers who quickly lost weight before competition [30].

Most studies have used adult athletes with significant sports experience, who have anxiety and stress derived from competition. However [9] studied 97 young judoka of both sexes during the Spanish national judo championship (age: 14.7 ± 1.3, range: 13–16) and their results demonstrated high somatic and cognitive anxiety as well as higher values when the judoka were younger (U-14 vs. U-16). 12 male judokas were studied [31] (age: 22.2 ± 1.6) at the regional and interregional levels and similarly discovered cognitive and somatic anxiety, but these anxieties were higher at the interregional levels (higher level judoka vs. regional-level judoka).

There is minimal scientific literature in this field, with no follow-up over different competitions for athletes of a young age, and depending on the importance of the competition, differences in stress and how stress affects them. However, we believe that there are differences between different competitions in the different variables, depending on the moment of the season.

For this reason, it is necessary to raise awareness among young athletes of the negative effects of weight loss linked to dehydration on physique and mental health, and the possibility of reaching states of malnutrition over time. The aim of this study was to analyze and compare the variation of the variables in three moments and determine the differences in bodyweight, hydration levels, and lean and fat mass between different competitions over time in order to create and design future strategies for the training of young athletes, to ensure their well-being and success, not only in their sports performance, but also in their health and correct growth and development.

## 2. Materials and Methods

### 2.1. Participants

Twenty-two elite judoka men (N = 22) were selected for this study. For various reasons, 2 subjects were lost. Ultimately, the sample was N = 20, (age 18.05 ± 1.05 years old, bodyweight 74.40 ± 9.71 kg, height 1.74 ± 0.05 m, BMI 24.39 ± 2.88, kg/m^2^ hydration levels 63.43 ± 3.02 Percent) (Table 2).

All the judoka belonged to the junior category, and the athletes trained at the high-performance center of the Higher Council of Sports Center of Spain. These athletes also belonged to the Detection of talents program of the Spanish Judo Federation, training at least three times a week in the morning.

Informed consent was sent to the athletes’ parents with an explanation of the procedures that were followed in the research. It was also necessary that the judoka had gone to the bathroom and that they had not consumed alcohol in the last 8 h. We also checked that there were no skin lesions in the areas contacted by the electrodes and that the judoka did not have any metallic implants.

This study was conducted in accordance with the Declaration of Helsinki, and our protocol was approved by the Ethics Committee of Universidad Politécnica de Madrid with the register number 2018-049/2018-050.

### 2.2. Procedure

The study design is observational, analytical, populational, transversal, and the investigation was explained with a written description. On the first day, the athletes were familiarized with the protocol. Prior to this project, a pilot study was conducted with the judoka to validate the protocol and eliminate reactivities, as well as increase the protocol’s reliability and validity. The adapted protocol [32] was given to the subjects before every test that was going to be measured. This protocol required total sincerity and was developed to obtain useful information and to control the variables of confusion that could damage the data from the investigation. In the following days, it was explained how the instruments functioned. Before each weighing, the participant was subjected to a series of questions in which the use of diuretic substances was controlled; previous evacuation, breakfast, wounds or burns interposed in the passage of the electricity generated by bioimpedance, or the use of metallic elements during weighing. All measurements were made in the morning in a changing room where the participants were weighed with privacy.

Once the measurements were started, all subjects were weighed under the same conditions, after having breakfast (a necessary condition before training so that the athletes had a high enough energy intake) from the High-Performance Sport Centre.

The athletes were required to control their weights pre and post competition (24 h before and 48 h later) during four different championships or leagues to determine the changes in their weights, hydration levels, lean mass, and fat mass over time.

**Figure 1:**
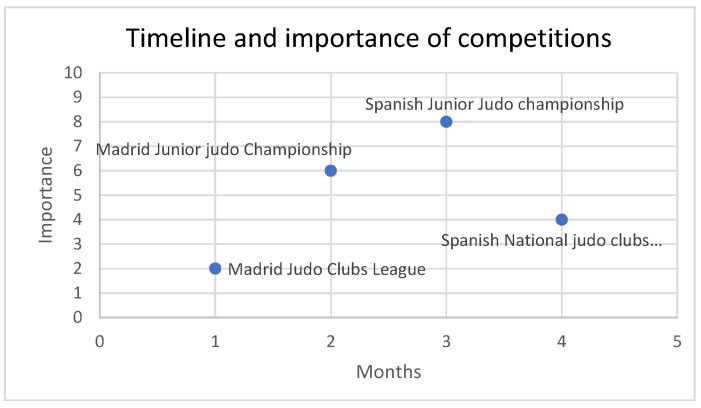
Timeline and importance of competitions during the season.

The judokas were measured after the Madrid Junior Judo Championship (1), before and after the Madrid Judo Clubs League (2), before and after the Spanish Junior Judo Championship (3), and before the Spanish National Judo Clubs League (4) during 4 months (January to April). These competitions are explained below with the importance degree (+/++++) following how they were scheduled during the season across the time (Figure 1).

(1) The Madrid Junior Judo Championship (MJJC) is a championship where the judoka fight to classify for the Spanish Judo Championship; this is a high importance competition (+++) it gives place to the Spanish National Judo Championship, in addition to regional scholarships and sports promotion. With an average of 3 rounds.

(2) The Madrid Judo Clubs League (MJCL): The Madrid League is organized by clubs, with three rounds and is the least importance competition (+).

(3) The Spanish Junior Judo Championship (SJJC) is a national championship where the judoka fight to be the individual Spanish junior champion; this is the competition with the highest importance (++++) and average of 2 rounds.

(4) The Spanish National Judo Clubs League (SNJCL): A national league with three rounds (the third round only occurs if the teams qualify); this is the competition with the second lowest importance (++).

The instrument used was a Tanita Inner scan BC545N (Tanita Corp., Tokyo, Japan) auto calibrated, with a maximum weighing of 150 kg and an error of 100 grams to obtain the BIA (bioimpedance) and determine the variables of body weight (kilograms), hydration (% of water with respect to the Bw), and BMI (kg/m^2^) [33,34,35] without taking into account the bone mass also collected by Tanita M ± 2.5 kg, which remain stable. 

Also was used Microsoft Excel v.2016 software by Windows (Microsoft, Redmond, Washington, EE.UU) to sort the variables and record the data in a spreadsheet, and IBM SPSS statistics software v.25 (IBM Corp. Released, Armonk, NY, EE.UU) was used for statistical analyses. In this study we look at three phases. These phases, called evaluation moments. Show us how the variables evolve to the abrupt weight loss of the competitors. 

These three moments were:

1. First moment: The Madrid Junior Judo Championship (MJJC+++) and Madrid Judo Clubs League (MJCL+),

2. Second moment: Madrid Judo Clubs League (MJCL+), Spanish Junior Judo Championship (SJJC++++),

3. Third moment: Spanish Junior Judo Championship (SJJC++++) and Spanish National Judo Clubs League (SNJCL++),

To simplify the analysis, all the variables were changed into kilograms and measured to show their descriptions at the different moments before and post competition. (Table 3).

### 2.3. Data Analysis

All data analysis was performed using the statistical software IBM SPSS statistics v.25. The standardized measures have been obtained from the variables analysed, using the mixed linear model procedure. Mixed linear models describe the relationship between bodyweight, hydration levels, and BMI variables and the different moments of measurements. This statistical procedure has been done to avoid possible interferences in p values when performing a null hypothesis test. Since this approach can be confusing according to the magnitude of the statistics, the measurement error or the sample size [36] was been used as a confidence interval, lower control limit (LCL), and upper control limit (UCL), that define the probable range of the true values. This difference between the positive and negative values that was obtained, were softened and graduate knowing that the true value obtained is the observed magnitude, (trivial/small (not significant) or moderated and large differences (significant)). Austin [37] found that a standardized difference of 10% (0.1) is equivalent to having a *p* > 0.05 (negligible correlation). 

## 3. Results

The results for the body weight variable show a higher mean body weight (kg) at post Madrid Junior Judo Championship MJJC +++ (the first competition) than in the pre measurement M (kg): 74.7 [70.4–78.9]–73.5 [69.2–77.8] and the third moment (SJJNC ++++–SNJCL ++) M: 74.9 [70.6–79.2]–74.3 [70–78.6], opposite to the second moment (MJCL +-SJJNC ++++) M: 73.9 [69.6–78.1]–74.4 [70.1–78.6], being a higher M pre than post competition SJJNC ++++, may be for the strict rules without margin of 1 kg of weight (as in competitions 1 and 2 (MJJC +++-MJCL +), led by the Madrid judo federation), in this case, set by the Spanish Judo Federation in competitions 3 and 4 (SJJNC ++++ – SNJCL ++) (Table 4)

The standardized difference in variable bodyweight, comparing Madrid Junior Judo Championship (MJJC) and Madrid Judo Clubs League (MJCL), has been not significant being the body weight higher at the first measure MJJC. The differences in bodyweight comparing moments were not significant (1 MJJC–3 SJJNC, 2 MJCL–3 SJJNC, and 2 MJCL–4 SNJCL) and significant differences between (1 MJJC–4 SNJCL and 3 SJJNC–4 SNJCL) (Table 5).

The variable hydration levels are balanced across the different moments M (kg between beginning and end): 47.4 [44–50.9] (1), 47.4 [44.9–49.9] (2), and 47,8 [45.1–50.6] (3) (Table 6).

The standardized differences obtained in the variable hydration levels comparing the moments showed higher levels of hydration in the previous competitions with which they were compared, there were significant differences in all comparisons, except between moment 2 MJCL+ and 4 SNJCL++ (0.18 trivial differences), which had significant differences. It can be inferred that the hydration levels at time 1 MJJC +++ were moderately different and superior to those at time 4 SJJNC ++ [range: 0.16–1.53]. The standardized difference obtained in the same variable, comparing moments 2 and 3 (MJJCL + and SJJNC ++++), the difference was significant, with hydration being greater at MJJCL + (Table 7).

The standardized difference obtained in the BMI variable comparing moments 1–2 (MJJC+++- MJCL +) was not significantly small, being higher in moment 1 MJJC +++. The standardized differences obtained in BMI compared to the other moments, showed a higher BMI in the later competitions, with significant differences * (0.21/0.55/0.37/0.71) (1 MJJC +++ – 3 SJJNC ++++, 1 MJJC+++–4 SNJCL +++, 2 MJCL +–4 SNJCL +++, 3 SJJNC ++++–4 SNJCL +++) and not significant differences (2 MJCL +–3 SJJNC ++++) (0.16 small), aside from affirming that it is the moment, where it can be inferred that the true value has the magnitude of the observed value [LCL −1.32 – UCL −0.10] so BMI was higher in the last competition (4 SNJCL +++–2 MJCL +) (Table 8).

In the fat mass variable (Table 9), there are no important differences in the fat mass between judokas at *p* = 0.256 or over time during the different moments *p* = 0.631. Therefore, variability in bodyweight is not determined by the gain or loss of fat mass since there are no difference. 

## 4. Discussion

The main goal of this study was to analyze and compare the variation of the variables in three periods and determine the difference in the bodyweight, hydration levels, and lean and fat mass for different competitions over time among junior judoka. In these three phases, we can see how the variables behave against the aggression that involves the sudden drops in the competitors.

Periods: first moment (MJJC+++and MJCL+), second moment (MJCL+ and SJJC++++), and third moment (SJJC++++ and SNJCL++).

The practices of food restriction and hydration restriction are well known in combat sports such as judo, taekwondo, boxing, and wrestling [38,39]. The athletes participating in this study reported problems in meeting their weight goals for weigh-in day (only a few days before weigh-in).

**Figure 2:**
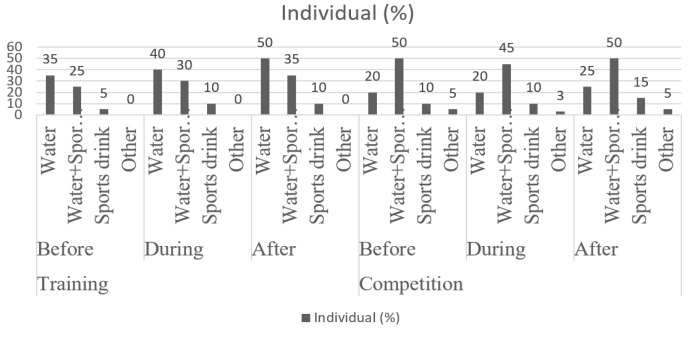
Hydration guidelines before and after training and competition for athletes of combat modalities (taekwondo, judo, and boxing). Adapted [2]. Water/Water + sport drink/sports drink/Other

The first weigh-in is done after the first competition in order to see the weight drop to enter category. This supposes a great effort for the organism. Once the competitors are subjected to weighing, they stop worrying about the weight and then competitive anxiety, due to the trauma of losing weight, added to the effect of the bodys own compensation, produces a rebound, weight gain reaction. These young athletes are habituated to receive a few hydrations breaks during training but are not encouraged to drink by the team. It is also necessary to understand (as outlined in previous studies [2]) the guidelines for hydration and the habits (Figure 2) necessary to maintain an adequate level of bodily fluids for combat athletes before, during, and after training and competition.

We collected our data during different moments pre and post competition from a large sample of athletes training in the High-Performance Sport Centre. Moreover, the judokas were randomly selected to participate, and their rate of acceptance was relatively high.

The results show that there are differences between hydration levels, which are more marked in the first week and less marked at later points in time, but there is a significant difference in water levels over time. The main findings of this study are the marked and continuous decrease during all the measurements. Especially during the first moment, dehydration affected body weight. This means that the differences in bodyweight during the different moments exist and are significant.

The marked variations in body weight and dehydration levels in the judoka sample agree with the ~60% of judo athletes that started rapid weight loss at the ages of 12–15 (M age: 18.05) as well as those in other combat sports such as karate and taekwondo, for whom the reported beginning of these procedures occurs around 13.6–14.2 years [1]. Others studies like [40] also showed, in a large sample of judoka female and male between 12 and 46 years old, that 86% of athletes resorted to rapid weight loss before competition and cut around 5% of their bodyweight; this study showed that at 15 years old, judoka began cutting weight using a combination of dehydration inducing methods including excessive exercise and a restriction of food. This past study also showed that cutting body weight to also lose water weight can be dangerous. These results could be useful for future research on controlling diet and improving performance and results in competition and to determine how these practices affect performance. 

Study [41], which evaluated 56 wrestlers during a wrestling championship, of that two groups emerged, hydrated vs. dehydrated. It was found that there were significant differences between both groups in the levels of cortisol (higher levels in dehydrated group) and testosterone (higher levels in hydrated group). As the results existing literature prove that these practices of weight loss and dehydration in all categories, the early ages at which they already begin to take place worrying.

On the other hand, there are no difference between the lean mass and fat mass in the subjects over time. Fat mass remain stable, while the athletes tried to reduce their weight through variables such as hydration, but not musculature, which is responsible for producing strength in combat; the goal of this process is to optimize performance [2].A study is carried out to determine the effects of large and sudden increases in training volume on performance characteristics and the overtraining syndrome symptoms among 15 elite judo athletes through 10 weeks of training suggests that, despite their training weeks, the athletes’ body weights did not change from weeks 2 to 10 [19]. This supports the idea that, since all junior elite male judoka train at the same high-performance center and with the same federation technicians, no difference can be explained because they follow the same training routine [3]. However, we should comment on the higher fat mass levels during the first moments of the Madrid Junior Judo Championship (1) MJJC +++ that was the first competition after the pre-season. There are several studies that indicated that body fat levels are higher before starting and preseason. In order to reduce energy intake and body fat percentage, in addition to setting the weight category for the season (if necessary), one must adjust his or her techniques and tactics, aerobic conditioning, and strength training [1].

Considering the moments, the different levels, and importance of competitions, it is important to understand that the first competition of the Madrid Junior Judo Championship MJJC +++ (1) occurred after the pre-season, in which the athletes did not have a restricted control of their diet or body weight; at this stage, the difference between the pre and post weight was 1.2 kg. This indicates greater variability, like in Madrid Judo Clubs League MJCL + (2), in which a common practice detected was to fight in higher weight categories than their usual competition weight. It supposes not previous control of the corporal weight, being above, in addition to the added dehydration. All these practices, adding high intensity training for rapid weight loss, cause a decrease in the immune system [16,42] The Spanish Junior Judo Championship SJJNC +++ (3), the most important competition of the season which possibly indicates a higher body weight for an athlete to reduce the psychological pressure [38,39,40,43] of his weigh-in and to arrive as close as possible to his weight-category [1,40]. In addition, competition produces levels of stress, already studied by authors [33], who measured and compared salivary cortisol [44] and salivary immunoglobulin responses between simulated and official jiu jitsu matches. The salivary cortisol was lower during simulated matches compared with that during official matches and lower at premeasurement when compared with post measurements, suggest that the high physical-physiological demands from official jiu jitsu and combat sports matches maximized stress hormone responses. The same as in to the first situation, after an aggressive and abrupt descent, the rebound effect occurs [6]. This apparently does not worry the competitor because in the next competition, they should not lose so much weight (SNJCL ++), competing in a higher category.

Regarding body water, all the measures indicated lower water levels before competitions.

For practical implications, it is important advice to all athletes to drink enough fluids all day to avoid a dehydrated state, both in training and in competition. These young athletes experienced dehydration during different moments, mostly during the first week in order to transition from the holidays and pre-season to the first competition of the year. Adolescents and lower category athletes must be encouraged to drink before, during, and after practicing any activity, and the team responsible for them should observe and control their hydration status and index. Of course, control and evaluations must be individualized and should replenish the fluids lost during judo activity. Furthermore, athletes should frequently avoid the common practice of restricting fluid intake and engaging in rapid weight loss strategies to reduce and eliminate the negative effects of marked dehydration on mental and physical health and performance, and guide sports practice to integral development [7,45,46,47].

## 5. Limitations

Possible limitation may be are low number of subjects, in fact, it is possible that some of the differences are not significant due to the low number two subjects were lost during the development of the study and data presented a great dispersion. The dimensions of the country and the rest of the High-Performance Sport centers were also considered for this study, but a sample from all centers would be impractical.

## 6. Conclusions

The results in this paper show that bodyweight, hydration levels, and BMI were generally higher after competing, than before competing, perhaps because of the immediate recovery to which athletes submit to minimize fatigue and psychological stress of weight loss prior to competitions. Except in the Spanish National Judo Clubs League SNJCL ++, where the level of hydration was slightly higher before the competition. So, it can be said that if they used weight loss strategies, and consequently a loss of water and BMI.

The bodyweight changes were not significant, except between the first and the last competition, being national tournaments.

There are no differences between the fat free mass and fat mass.

The standardized differences in BMI changes have been significant, except between Madrid Junior clubs league MJCL + and Spanish National Junior Judo Championship SNJJC++++, where they have been not significant due to their temporary proximity.

The differences in hydration are significant. 

The training types are linear and undulating training does not seem to make a difference in performance. Thus, some sessions with an aerobic part could be organized during the competition period, where coaches could organize sessions with strength and speed as the dominant theme, showing that undulating training could allow a few aerobic sessions during the competition period to facilitate weight loss [48]. For implications, can be interesting try different training methods and measure the different variables and performances.

Considering the established objectives and our results, we conclude that a decrease in hydration levels produces a difference in bodyweight, junior judokas are involved in weight loss methods compromise their hydration and health before competitions. Being, interesting create and design futures strategies for the training of young athletes to ensure their well-being and success, not only in their sports performance, but also in their health and correct growth and development.

## Figures and Tables

**Table 1:** Percentage of body fat (mean M and standard deviation ±SD) of national and university teams according to the measurements of different authors and years.

Nationality	% of Body Fat		Author and Year
	M	±SD	
Olympic North American Judo team	8.3	1.0	Callister et al. (1991) [19]
Japan university judo team	16.2	5.7	Iida et al. (1998) [20]
Turkish national judoka	9.5	0.9	Osman Imanoglu et al. (1999) [21]
Brazil university judo team	13.7	5.2	Franchini et al. (2005) [7]
Indian judoka >5 years/practice	13.8	6.4	Jayasudha Katralli et al. (2012) [22]

Adapted [23,24]. The data are presented as the mean (M) (±SD) and range.

**Table 2:** Characteristics (descriptive statistics) of the judoka’s ages, bodyweights, height, body mass index hydration levels, and bodyfat.

	Age (years)	Body Weight (kg)	Height (m)	BMI	Hydration (% Water/Bw)	Lean Mass (kg)
N = 20	18.05 ± 1.05	74.40 ± 9.71	1.74 ± 0.05	24.39 ± 2.88	63.43 ± 3.02	60.88 ± 6.46

Data are presented as the mean (M) and (±SD).

**Table 3:** Characteristics of the judokas at the different moments (mean M and standard deviation ±SD) post and pre competition in body weight, water levels, lean mass, and fat mass.

			Body Weight (kg)		Water Levels (kg)		Lean Mass (kg)		Fat Mass (kg)	
		Moment	Post	Pre	Post	Pre	Post	Pre	Post	Pre
N = 20	Mean	1 MJJC-MJCL	74.7	73.5	48.4	46.5	61.1	61.0	10.9	10.3
		2 MJCL-SJJNC	73.9	74.4	47.4	47.3	61.0	61.4	10.2	10.0
		3 SJJNC-SNJCL	74.9	74.3	48.1	47.6	61.8	61.4	10.3	10.2
	Standard deviation	1 MJJC-MJCL	9.6	9.9	5.5	5.3	6.5	6.6	6.9	6.6
		2 MJCL-SJJNC	9.9	9.3	5.8	5.2	6.7	6.6	6.5	6.2
		3 SJJNC-SNJCL	9.3	9.4	5.7	5.9	6.5	7.1	6.0	6.0

The data are presented as the mean (M) (±SD) and range; 95% CI.

**Table 4:** Changes (mean M, standard error SE and range) among the different moments and comparing the pre and post competitions for the body weight variable (kg).

	Moment	Week Part	M	SE	Range	
Body weight	MJJC +++ – MJCL +	Post	74.7	2.14	70.4	78.9
		Pre	73.5	2.14	69.2	77.8
	MJCL + – SJJNC ++++	Post	73.9	2.14	69.6	78.1
		Pre	74.4	2.14	70.1	78.6
	SJJNC ++++ – SNJCL ++	Post	74.9	2.14	70.6	79.2
		Pre	74.3	2.14	70.0	78.6

The data are presented as the mean (M) and standard error (SE) and range; 95% CI, *p* = 0.05.

**Table 5:** Standardized differences of the variable analyzed bodyweight at the different moments: The Madrid Junior Judo Championship (MJJC) (moment 1), Madrid Judo Clubs League (MJCL) (moment 2), Spanish Junior Judo Championship (SJJC) (moment 3), and Spanish National Judo Clubs League (SNJCL) (moment 4).

	Moments		Standardized Differences of the Means	LCL	UCL
Changes in Bodyweight	1+++	2+	0.05	−0.53	0.63
	1+++	3++++	−0.14	−0.72	0.44
	* 1+++	4++	−0.26	−0.84	0.32
	2+	3++++	−0.05	−0.77	0.39
	2+	4++	−0.19	−0.89	0.27
	* 3++++	4++	−0.31	−0.70	0.46

The data are presented as standardized differences and Lower Upper levels; 95% CI. Competitions are explained with the importance degree (+/++++). (* significant differences)

**Table 6:** Changes (mean M, standard error SE and range) among the different moments and comparing the week’s beginning and end in the water levels variable (kg).

	Moment	Week Part	M (Kg)	SE (Kg)	Range (Kg)	
Water Levels	1 MJJC +++	Beginning	48.4	1.25	45.9	50.9
		End	46.5	1.25	44.0	49.0
	2 MJCL +	Beginning	47.4	1.25	44.9	49.9
		End	47.3	1.25	44.8	49.8
	3 SJJNC ++++	Beginning	48.1	1.25	45.6	50.6
		End	47.6	1.25	45.1	50.1

The data are presented as the mean (M) and standard error (SE) and range; 95% CI, (*p* = 0.05). Competitions are explained with the importance degree (+/++++).

**Table 7:** Standardized differences of the variable analyzed hydration levels at the different moments: Madrid Junior Judo Championship MJJC+++ (moment 1), Madrid Judo Clubs league MJCL + (moment 2), Spanish Junior Judo Championship SJJNC++++ (moment 3) and Spanish National Judo Cubs league SNJCL++ (moment 4).

	Moments		Standardized Differences of the Means	LCL	UCL
Differences in Hydration levels	* 1 MJJC +++	3 SJJNC ++++	0.51	−0.18	1.19
	** 1 MJJC +++	4 SNJCL ++	0.85	0.16	1.53
	* 2 MJCL +	3 SJJNC ++++	−0.33	−0.51	0.86
	2 MJCL+	4 SNJCL ++	0.18	−0.17	1.20
	* 3 SJJNC ++++	4 SNJCL ++	0.52	−0.35	1.02

The data are presented as the standardized differences, lower and upper levels; 95% CI. Competitions are explained with the importance degree (+/++++). (* significant differences (moderate) ** significant differences (large))

**Table 8:** Standardized differences of the variable analyzed body mass index at the different moments: Madrid Junior Judo Championship (MJJC) ++ (moment 1), Madrid Judo Clubs League (MJCL) + (moment 2), Spanish junior judo championship (SJJNC) ++++ (moment 3) and Spanish National Judo Clubs League (SNJCL) +++ (moment 4).

	Moments		Standardized Differences of the Means	LCL	UCL
Differences in BMI	1 MJJC +++	2 MJCL +	0.16	−0.44	0.77
	* 1 MJJC +++	3 SJJNC ++++	−0.21	−0.82	0.39
	* 1 MJJC +++	4 SNJCL +++	−0.55	−1.15	0.06
	2 MJCL +	3 SJJNC ++++	−0.16	−0.98	0.23
	* 2 MJCL+	4 SNJCL +++	−0.37	−1.32	−0.10
	* 3 SJJNC ++++	4 SNJCL +++	−0.71	−0.94	0.27

The data are presented as the standardized differences, lower and upper levels; 95% CI. Competitions are explained with the importance degree (+/++++). (* significant differences)

**Table 9:** Mean difference M standard deviation ± SD and range comparing the different moments in the fat mass variable (kg).

	Moment Comparisons	M (kg)	SE (kg)
Fat Mass	Moment 1 Madrid Junior Judo Championship MJJC +++ Moment 2 MJCL +	0.49	1.99
	Moment 1 Madrid Junior Judo Championship MJJC+++ Moment 3 Spanish Junior Judo Championship SJJNC ++++	0.34	1.99
	Moment 2 Madrid Junior Clubs League MJCL + Moment 3 Spanish Junior Judo Championship SJJNC ++++	−0.16	1.99
	Moment 3 Spanish Junior Judo Championship SJJNC ++++ Moment 4 Spanish National judo clubs league SNJCL ++	−0.25	1.99

The data are presented as the mean (M) and standard error (SE). Competitions are explained with the importance degree (+/++++).
